# Identification of MAEA protein as a potential target for chemoresistance in osteosarcoma using bioinformatics and proteomic analysis

**DOI:** 10.3389/fonc.2025.1597750

**Published:** 2025-09-08

**Authors:** Chen Zhang, Ruizhen Wang, Xin Yi, Wannian Wang, Jing Yang, Lihua Zhang, Guibin Wang, Wei Wang

**Affiliations:** ^1^ Senior Department of Orthopedics, The Fourth Medical Center of PLA General Hospital, Beijing, China; ^2^ Department of Central Laboratory and Mitochondrial Medicine Laboratory, Qilu Hospital (Qingdao), Cheeloo College of Medicine, Shandong University, Qingdao, China; ^3^ Academy of Chinese Medical Sciences, Henan University of Chinese Medicine, Zhengzhou, Henan, China; ^4^ Department of General Surgery, Qilu Hospital (Qingdao), Cheeloo College of Medicine, Shandong University, Qingdao, China; ^5^ Department of Animal Genetics, Breeding and Reproduction, College of Animal Science, Shanxi Agricultural University, Taigu, China; ^6^ Senior Department of Ultrasound, The First Medical Center of PLA General Hospital, Beijing, China; ^7^ Senior Department of Pathology, The Fourth Medical Center of PLA General Hospital, Beijing, China; ^8^ State Key Laboratory of Medical Proteomics, Beijing Proteome Research Center, National Center for Protein Sciences (Beijing), Beijing Institute of Lifeomics, Beijing, China

**Keywords:** osteosarcoma, proteomic, chemoresistance, biomarker, clinical

## Abstract

**Introduction:**

Osteosarcoma (OS) is the most common bone tumor, characterized by a high incidence, rapid progression, and frequent metastases. The implementation of chemotherapy has made important progress, while the necrosis rate is limited and the survival rates remain unsatisfactory, therefore novel approaches are needed.

**Methods:**

We used proteomic analysis to characterize the molecular landscape of patients exhibiting different levels of chemotherapy-induced necrosis.

**Results:**

Patients with low necrosis rate (≤70%) showed distinct expression patterns, with significant upregulation of proteins involved in DNA replication, metabolism, and mitochondrial pathway. The Runx1-related signaling pathway was also identified as potentially involved in disease progression. Remarkably, Mitochondrial Ribosomal Protein L4 (MRPL4) and Macrophage Erythroblast Attacher, E3 Ubiquitin Ligase (MEMA) were identified as hub proteins in MEGENA analysis and the public database. By integrating with immunohistochemistry, the higher expression level was verified in samples of OS patients compared to those of healthy people.

**Discussion:**

Overall, our project improves the knowledge of the expression pattern with different necrosis rates of OS samples, and the findings of MRPL4 and MAEA indicate the potential role in chemoresistance and provide new targets for the therapeutic strategy for OS patients with a low necrosis rate.

## 1 Introduction

Osteosarcoma (OS) is the most common bone tumor, with the global annual incidence of approximately three to four per million (1). It most commonly affects adolescents and young adults, with a secondary incidence peak occurring in adults aged 65 years and older (2). Typical symptoms include pain and swelling in the affected bones, most frequently in the metaphyses of long bones (3). Occasionally, patients present with severe pain strong enough to awaken them from sleep or with signs associated with a pathologic fracture (4). Meanwhile, approximately 15% to 20% of patients present with clinically detectable metastases (5). The aggressive nature of OS, characterized by its high metastatic potential and rapid progression, poses significant clinical and socioeconomic challenges.

A variety of chemotherapy regimens have been used to treat OS since chemotherapy was introduced more than 40 years ago. A combination of methotrexate, doxorubicin, cisplatin, ifosfamide, and etoposide has demonstrated efficacy in patients (6). However, the outcome for OS patients remains unsatisfactory, mainly ascribed to the development of resistance to chemotherapy (7). This dilemma highlights the need for a novel approach, in combination with standard chemotherapy, to improve outcomes and survival rates in patients.

Remarkably, in the initial trials assessing good- and poor-responder groups, the > 90% 5-year event-free survival rate was identified in the group with > 90% necrosis after neoadjuvant chemotherapy (8). Comparatively, for patients with poor histologic response, considered to be less than 90% necrosis, the 5-year survival was only 50% to 60% (9). Therefore, chemotherapy-induced necrosis positively correlates with survival in patients with high-grade localized OS. This highlights the need to identify differences between groups with varying necrosis rates, which could help uncover potential mechanisms and inform combination treatments for patients with poor chemotherapy response.

Recent advances in proteomic profiling have provided critical insights into the molecular landscape of OS, leading to the identification of candidate biomarkers associated with tumor progression and response to chemotherapy. However, the number of proteins consistently shown to be differentially expressed remains limited, particularly when analyses are based on archived clinical specimens (10). In efforts to identify biomarkers predictive of chemotherapy response, two independent studies employed two-dimensional difference gel electrophoresis (2D-DIGE) and liquid chromatography-tandem mass spectrometry (LC-MS/MS) (11, 12). While the specific protein profiles varied between studies, both identified peroxiredoxin 2 (PRDX2) as being upregulated in patients who exhibited poor responses to chemotherapy compared to good responders, suggesting its potential role in chemoresistance. Furthermore, the application of proteomics to formalin-fixed, paraffin-embedded (FFPE) OS tissues remains limited (13), significantly hindering the reproducibility and depth of protein identification. Consequently, there remains a substantial unmet need for robust and clinically applicable biomarkers in this setting.

Here, by combining proteomic analysis with immunohistochemistry, the main objective of our study was to explore the difference in the molecular landscape between patients with various necrosis outcomes, therefore providing a basis for downstream treatment.

## 2 Materials and methods

### 2.1 Patient selection

The cohort included 29 OS patients who underwent chemotherapy in the Senior Department of Orthopedics, Fourth Medical Center of the PLA General Hospital, from January 2020 to March 2024. Patients were selected according to the following criteria: (i) historically confirmed diagnosis of OS; (ii) receipt of at least three cycles of chemotherapy before surgery; and (iii) availability of tumor tissue fixed in formalin and complete medical records, including age, gender, subtype, pathogenical site, and necrosis rate. The control group comprised 10 patients with osteoarthritis, aged 50–70 years, who underwent total knee replacement surgery and had histological specimens, with no previous history of cancer. All experiments were approved by the ethics committee of the Fourth Medical Center of the PLA General Hospital, and written informed consent was obtained from all patients.

### 2.2 Sample preparation

The FFPE (13) samples were soaked in xylene three times, in anhydrous ethanol twice, and in 75% ethanol once, then dried at room temperature (14). The tissue samples were scraped off with a clean blade, lysed with 100 µl of lysis buffer (1% sodium deoxycholate monohydrate [SDC], 100 mM Tris-HCL, 10 mM tris [20 carboxyethyl] phosphine hydrochloride [TCEP], 40 mM chloroacetamide [CAA], protease inhibitor), and subjected to protein qunatification using a BCA protein assay kit.

### 2.3 Protein digestion

Ten millimolar DTT was added to the protein extract and incubated at 56°C for 1 h. After cooling to room temperature, 55 mM IAA was added and incubated at room temperature in the dark for 45 min. Based on the protein concentration measurement results, an appropriate amount of protein was taken from each sample for the filter-aided sample preparation (FASP) enzymatic digestion method (15). For phosphorylation enrichment, 50 μg of peptides from each sample were enriched using the phosphorylation enrichment kit Fe-NTA (Thermo Fisher, 81 Wyman Street, Waltham, MA).

### 2.4 LC-MS/MS analysis

Solvent A for LC was prepared with 0.1% formic acid in water, while solvent B was prepared with 80% acetonitrile and 0.1% formic acid in water. Lyophilized peptides were dissolved in 0.1% formic acid in water and then centrifuged for 15 min at 17,000 × *g*. The resulting supernatant was injected into a C18 analytical column (150 μm × 25 cm) using an EASY-nLC 1200 HPLC (Thermo, USA) at a flow rate of 2 μL/min. Peptides were eluted from the analytical column at a flow rate of 600 nL/min with a gradient to 7% B at 0 min, 12% B at 10 min, 30% B at 57 min, 45% B at 79 min, and 95% B at 81 min, which was maintained until 90 min. The eluted peptide was sprayed at a voltage of 2.2 kV and analyzed using a Thermo Scientific Orbitrap Fusion mass spectrometer coupled with a Nanospray Flex ion source. The ion transfer tube was set at 320°C (16). The MS scan resolution was set to 120,000, the scanning range was set to 350–1,500 m/z, and the maximum injection time was set to 50 ms. The MS/MS scan resolution was set to 30,000, with 30 scanning windows. The scanning range for MS/MS was set to 200–1,600 m/z, the collision energy was set to 33%, and the maximum injection time was set to 54 ms.

### 2.5 Database searching for protein identification

Raw DIA data were searched against a preferred database using Spectronaut version 17 (Biognosys, No.1, West Huanhu No.2 Rd, Pudong, Shanghai, P.R.China). The digestion enzyme was set to Trypsin/P, with a maximum of two missed cleavages allowed. Carbamidomethylation of cysteine (C) was specified as a fixed modification, while methionine oxidation and protein N-terminal acetylation were set as a variable modification. For the phosphoproteome, phosphorylation of STY residues was set as a variable modification. The FDR was controlled at 1% at both the protein and peptide levels, and a minimum of one peptide per protein was required for identification. All other parameters were set to default (17). The human reference database was downloaded from UniProt.

### 2.6 Principal component analysis and clustering analysis

PCA was used to retain proteins in the dataset that contributed most to its variance (18). K-means clustering of all the identified proteins was performed using the R packages “factoextra” and “cluster”. Hierarchical clustering of all the identified proteins was performed using the R package with the complete method.

### 2.7 Construction and validation of the ANN model, identification of differentially expressed proteins, and bioinformatics analyses

According to the information on chemotherapy-induced necrosis rate, the samples were divided into a low necrosis rate group with ≤ 70% as the low group, a medium necrosis rate group with 70% < necrosis rate ≤ 90% as the medium group, and a high necrosis rate group with > 90% as the high group. DEPs between groups were identified using R software with Student’s *t*-test, and a *p*-value < 0.05 was considered statistically significant.

Functional enrichment analysis, including Metascape (https://metascape.org/gp/index.html#/main/step1) (19), WebGesalt (https://www.webgestalt.org) (20), g:Profiler (https://biit.cs.ut.ee/gprofiler/gost), and KOBAS (http://bioinfo.org/kobas/genelist/) (21), was performed for investigating DEPs. The threshold was set at *p* < 0.05.

The relationships among the DEPs were investigated and visualized with protein–protein interaction (PPI) networks via the STRING web server (https://string-db.org/) (22) and Cytoscape software (version 3.6.1).

### 2.8 MEGENA analysis

MEGENA analysis was performed using the R package “MEGENA” (23). By constructing planar filtered networks (PFNs), MEGENA can effectively identify coexpression networks. Subsequently, multiscale hub analysis (MHA) was used to identify highly connected hubs within each network. A sunburst plot was generated using the R package “sunburstR” to illustrate the relationships between networks. Gene modules in MEGENA were then intersected with DEPs of the low group and the high–medium group to filter potential signaling pathways and corresponding hub proteins.

### 2.9 Immunohistochemistry

Samples were fixed in 10% formalin and sectioned. Paraffin sections were routinely dewaxed, rehydrated, and subjected to antigen retrieval. The sections were then incubated in 3% hydrogen peroxide solution at room temperature, protected from light, for 25 min, followed by washing on a decolorizing shaker three times for 5 min each. The tissue was then uniformly covered with 3% BSA for blocking at room temperature for 30 min. The blocking solution was gently removed, PBS was added to the sections, and they were incubated with one of the following primary antibodies: anti-CBX5 antibody (1:500; GB11609 - 100, Servicebio, No. 388, East Lake New Technology Development Zone, Wuhan, Hubei, China), anti-MAEA antibody (1:500; GB111748 - 100, Servicebio), anti-MRPL58 antibody (1:500; GB114424 - 100, Servicebio), or anti-MRPL4 antibody (1:200; bs-17778R, Bioss, Greater Boston Area, Massachusetts). The sections were placed flat in a humidified chamber at 4 °C for overnight incubation. After washing and gently drying, the tissue was covered with the appropriate species-specific HRP-labeled secondary antibody and incubated at room temperature for 50 min. Freshly prepared DAB substrate solution was then applied to the marked area, and the nuclei were conterstained before further microscopy analysis.

### 2.10 Statistical analysis

GraphPad Prism version 10.0 was used for statistical analyses. Statistical differences between two groups were assessed using Student’s *t*-tests, while differences among multiple groups were analyzed by one-way analysis of variance (ANOVA) followed by Tukey’s significant difference test for *post hoc* analysis. Statistical significance was defined as *p* < 0.05. Pearson correlation analysis was performed to evaluate associations between two variables.

## 3 Results

### 3.1 Low necrosis rate group showing specific proteomic landscape

**Figure 1:**
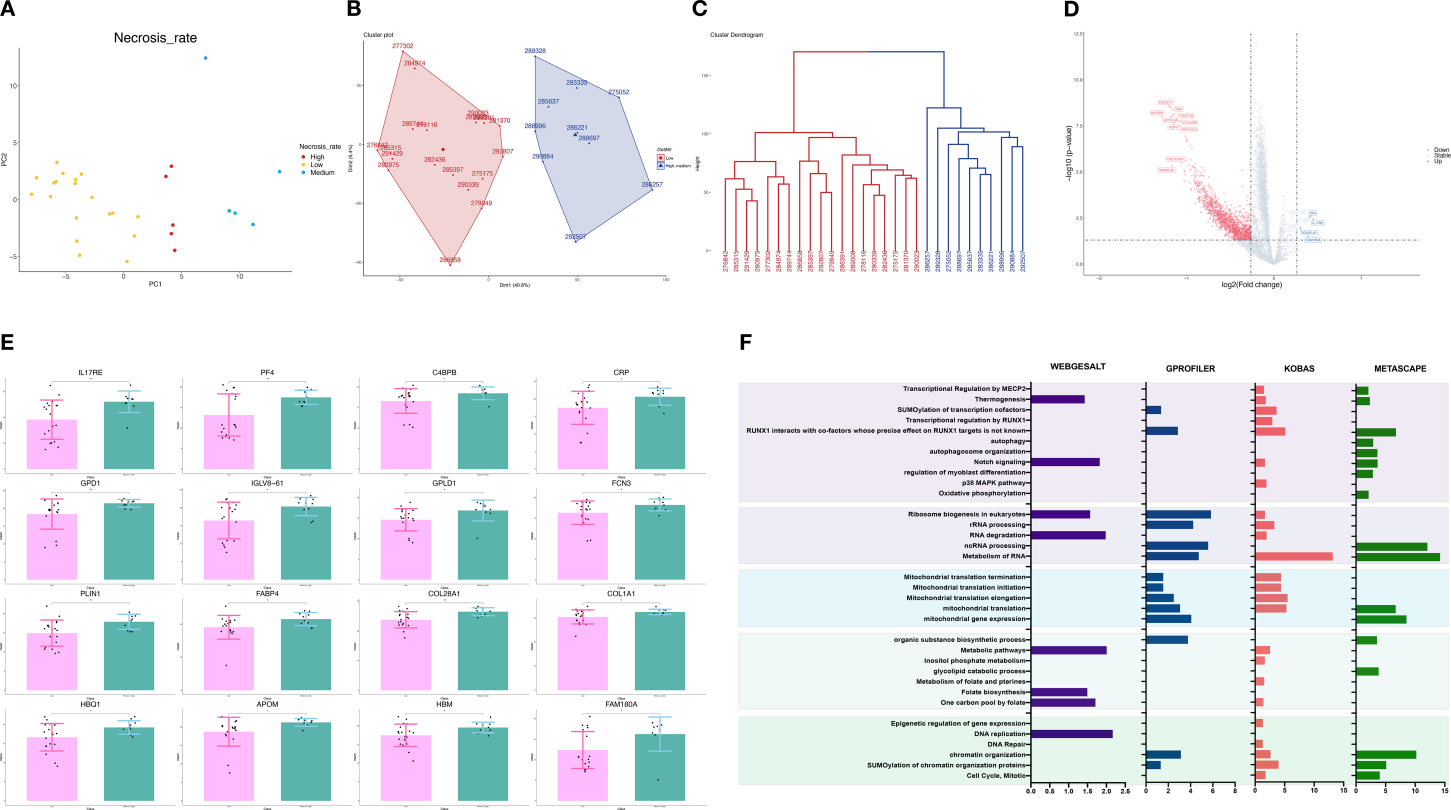
Proteomic profiling according to the necrosis rate in osteosarcoma samples. **(A)** PCA of proteomics data from 29 samples showing distinct separation by necrosis rate but not by gender, subtype, or tumor site. **(B, C)** K-means and hierarchical clustering highlighting differences between the low necrosis rate group (LNRg) and combined medium-high necrosis rate group (MHNRg). **(D)** Volcano plot of differentially expressed proteins between LNRg and MHNRg. Significantly upregulated proteins are highlighted in blue, and downregulated in red. Cut-off values are indicated by dashed lines. The *x*-axis represents log2 fold change. *y*-axis indicates −log10 (*p*-value). **(E)** Representative upregulated proteins in MHNRg (*N* = 10). **(F)** Pathway enrichment analysis of 200 LNRg-upregulated proteins using Metascape, WebGestalt, Kobas, and Gprofiler (*N* = 19). *x*-axis indicates −log10 (*p*-value). Statistical significance: ^**^ *p*-value < 0.01; ^***^ *p*-value < 0.001.

Proteomics data from 29 samples were included. Interestingly, principal component analysis (PCA) revealed that samples with different necrosis rates had distinct proteomic landscapes, in contrast to groupings by gender, subtype, or tumor site (**Figure 1A**, **Supplementary Figures S1A–C**). K-means and hierarchical clustering both demonstrated the difference in necrosis rates between the low the high/medium group (**Figures 1B, C**, **Supplementary Figures S1D, E**). Therefore, we integrated the medium (= 90%) and high (> 90%) groups as the medium-high necrosis rate group (MHNRg).

The volcano plot showed that the highly expressed and lowly expressed proteins could be clearly distinguished between low necrosis rate group (LNRg) and MHNRg (**Figure 1D**). The up-regulated proteins in MHNRg mainly included immune-related proteins (IL17RE, PF4, C4BPB, CRP, GPD1, IGLV8 - 61, FCN3, GPLD1), oxidant detoxification proteins (HBQ1, APOM, HBM), adipocyte lipid metabolism (FABP4, PLIN1) and collagen formation proteins (COL28A1, COL1A1) (**Figure 1E**). FAM180A, as one of the candidate proteins for personalized prognosis and tumor microenvironment phenotypes prediction in tumors (24), was highlighted as well.

In addition, there are more significantly expressed proteins in LNRg, indicating the occurrence of unique proteomic profiles in patients with an unsatisfactory response to chemotherapy. To further identify the specific characteristics, a total of 200 up-regulated proteins in LNRg were recruited for downstream pathway enrichment analysis. The analysis was performed using Metascape, Webgestalt, Kobas, and Gprofiler, revealing that DNA replication, metabolic, and mitochondrial-related pathways were enriched (**Figure 1F**). In addition, differentially expressed proteins (DEPs) in LNRg participate in classical signaling pathways such as Notch and MAPK, which have been reported to play an integral role in OS onset, progression, metastasis, and treatment response (25–27).

### 3.2 LNRg showing active metabolism

**Figure 2:**
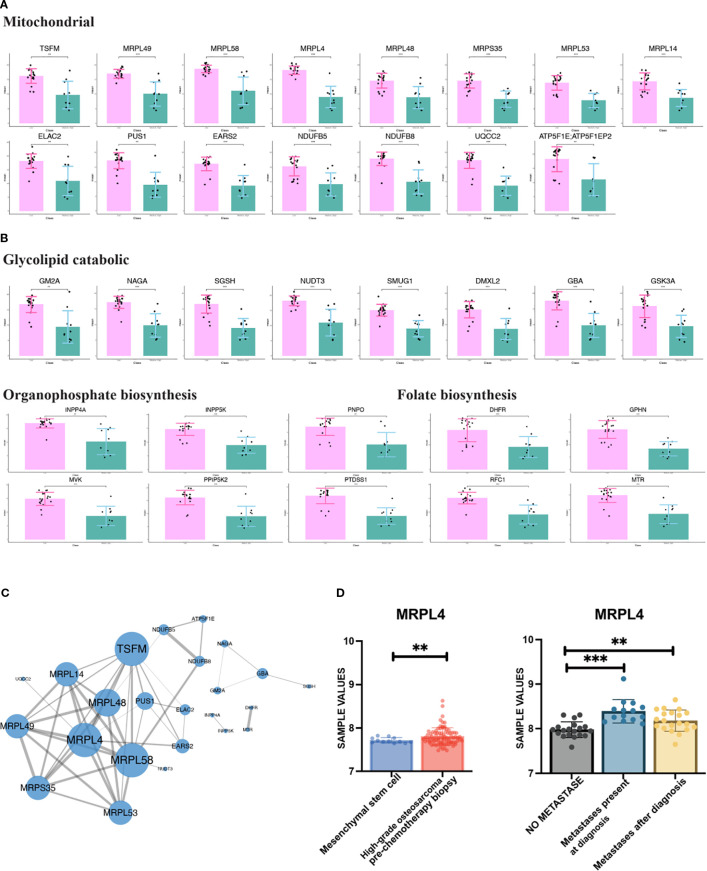
Metabolism-related differentially expressed proteins (DEPs) and their functional characterization in LNRg. **(A, B)** Enrichment analysis of upregulated DEPs in LNRg showing significant involvement in mitochondrial, glycolipid catabolic, organophosphate biosynthetic, and folate biosynthetic pathway. **(C)** Protein–protein interaction (PPI) network of metabolism-related DEPs constructed using STRING and visualized in Cytoscape. **(D)** Validation of MRPL4 expression in public datasets, showing higher expression in high-grade osteosarcoma compared to mesenchymal stem cells (MSCs) (left) and in metastatic versus nonmetastatic samples at both diagnosis and follow-up (right). Statistical significance: ^**^ *p*-value < 0.01; ^***^ *p*-value < 0.001.

Through enrichment analysis (**Figure 1F**), a substantial portion of up-regulated DEPs of LNRg was found to be related to metabolism. As illustrated in **Figure 2A**, GM2A, NAGA, SGSH, NUDT3, DMXL2, GBA, and GSK3A participate in the glycolipid catabolic process. The organophosphate biosynthetic process contains INPP4A, NPP5K, PNPO, MVK, PPIP5K2, and PTDSS1, while folate biosynthesis includes DHFR, GPHN, RFC1, and MTR. Meanwhile, 15 DEPs were associated with mitochondrial functions (**Figure 2B**), including mitochondrial translational elongation factor (TSFM), mitochondrial ribosomal proteins (MRPL49, MRPL14, MRPL53, MRPL58, MRPL4, MRPL48, and MRPS35), tRNA processing proteins (EARS2, PUS1, and ELAC2), and respiratory chain complex components (NDUFB5, NDUFB8, UQCC2, and ATP5F1E).

To further elucidate the functional interactions of metabolism-related DEPs, a comprehensive protein–protein interaction network was constructed using the STRING database, followed by visualization and hub module identification with Cytoscape (**Figure 2C**). Within this network, TSFM, MRPL4, and MRPL58 emerged as key hub proteins based on their central connectivity.

To further validate the potential hub genes, the public database (GSE21257 and GSE33383) was incorporated. It was shown that MRPL4 showed higher expression in samples of high-grade osteosarcoma than in mesenchymal stem cells (MSCs) as a normal control. Meanwhile, the expression of MRPL4 is higher in samples with metastasis, no matter at diagnosis and later, than those with no metastasis (**Figure 2D**). These results indicate the critical involvement of active metabolism, particularly the role of MRPL4, in osteosarcoma progression and metastasis, suggesting its potential as a biomarker or therapeutic target.

### 3.3 Runx1-related pathway activated in LNRg

**Figure 3:**
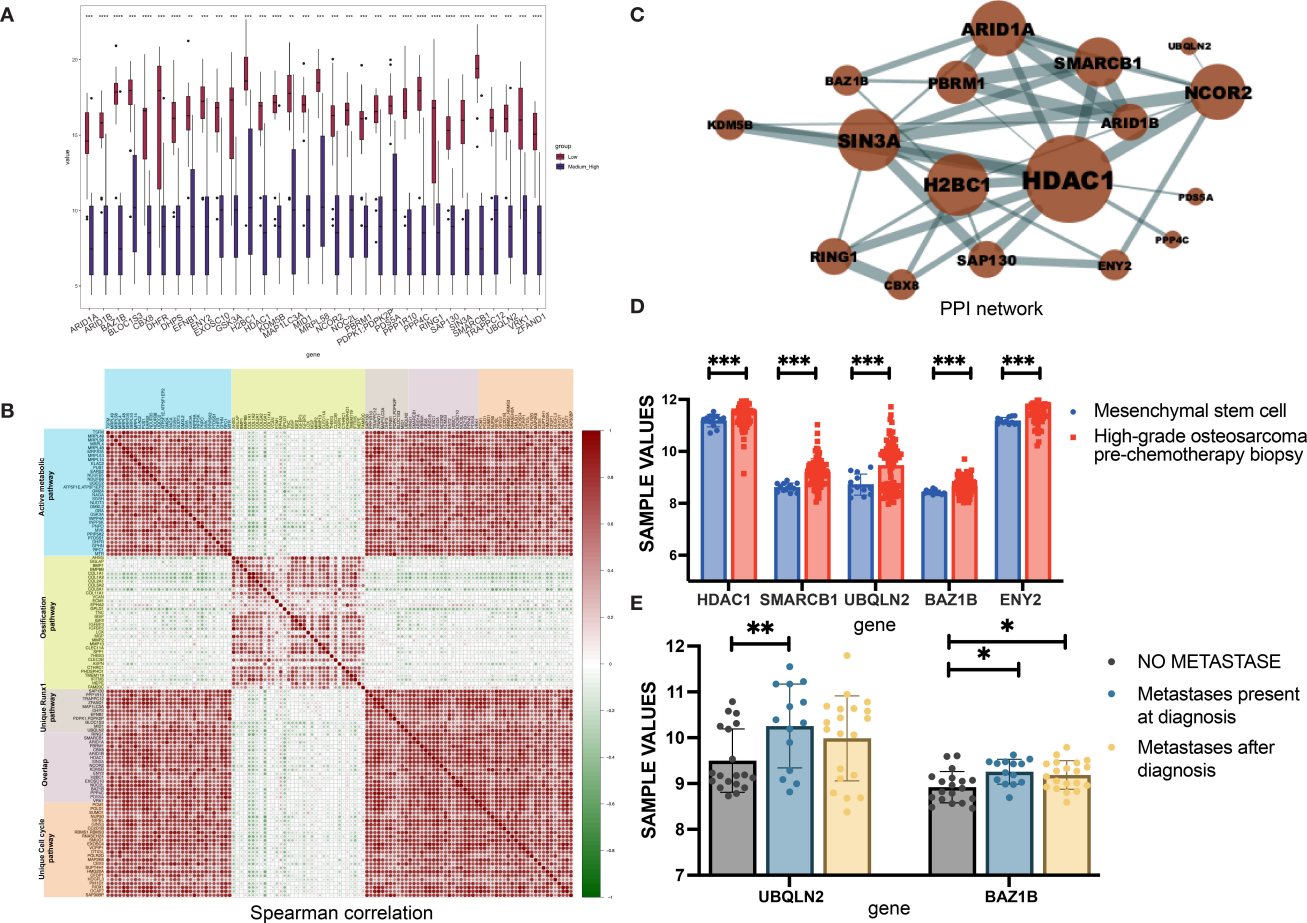
Identification of Runx1-associated molecular features in the LNRg. **(A)** Boxplots showing comparative expression levels of Runx1-associated proteins between the LNRg and the MHNRg. **(B)** Spearman correlation analysis illustrating relationships among proteins involved in active metabolism, ossification, Runx1 signaling, and cell cycle pathways. **(C)** PPI network depicting upregulated proteins associated with Runx1. **(D)** mRNA expression levels of HDAC1, SMARCB1, UBQLN2, BAZ1B, and ENY2 were lower in mesenchymal stem cells (MSCs) and prechemotherapy biopsies from high-grade osteosarcoma patients. **(E)** mRNA expression levels of UBQLN2 and BAZ1B in osteosarcoma samples categorized by metastatic status: without metastasis, with metastasis at diagnosis, and with metastasis after diagnosis. Statistical significance: ^*^ *p*-value < 0.05; ^**^ *p*-value < 0.01; ^***^ *p*-value < 0.001.

RUNX Family Transcription Factor 1 (Runx1)-related signaling was enriched in samples from the LNRg group, which comprises Ring Finger Protein 1 (RING1), SWI/SNF Related BAF Chromatin Remodeling Complex Subunit B1 (SMARCB1), AT-Rich Interaction Domain 1A (ARID1A), PBRM1, CBX8, ARID1B, Histone Deacetylase 1 (HDAC1), SIN3A, NCOR2, KDM5B, ENY2 Transcription And Export Complex 2 Subunit (ENY2), SAP130, H2BC1, EXOSC10, NOC2L, Bromodomain Adjacent To Zinc Finger Domain 1B (BAZ1B), PPP1R10, TRAPPC12, ZFAND1, MAP1LC3A, PPP4C, DHPS, EFNB1, PDPK1, BLOC1S3, PDS5A, MID1, Ubiquilin 2 (UBQLN2), and VRK1, all showing significantly higher expression levels than in the MHNRg group (**Figure 3A**). As an underexplored molecular pathway in OS, Runx1-related signaling pathway was identified to exhibit the expression pattern positively associated with active metabolic pathway and cell cycle-related pathway, while correlated with ossification pathway negatively (**Figure 3B**), indicating the potential role in disease progression.

To further identify the potential hub proteins in the Runx1-related pathway, we performed STRING analysis to build the protein–protein interactions. Seventeen DEPs were connected based on active interaction sources (**Figure 3C**). The larger the protein node, the more interactions it has. Notably, HDAC1 emerged as a hub protein, which was verified in the public database GSE33383 by comparing the expression level of MSCs with that of high-grade osteosarcoma prechemotherapy biopsy (**Figure 3D**). Meanwhile, higher expression levels of SMARCB1, UBQLN2, ENY2, and BAZ1B was observed in high-grade osteosarcoma prechemotherapy biopsy samples than that of MSCs. Furthermore, the comparative data between metastatic and nonmetastatic samples confirmed the role of UBQLN2 and BAZ1B in OS disease metastasis (**Figure 3E**).

### 3.4 Network enrichment analysis by MEGENA

**Figure 4:**
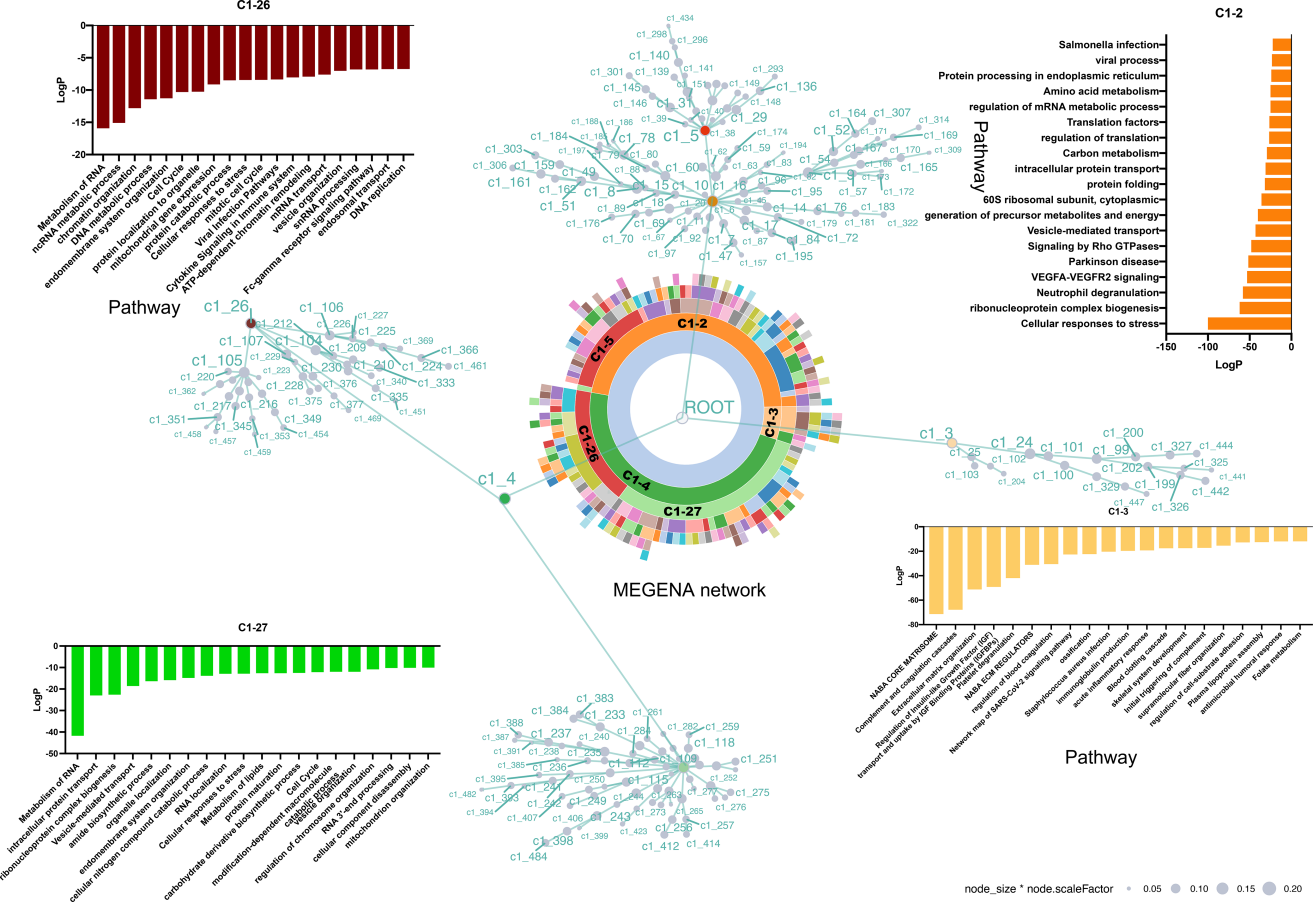
MEGENA network enrichment analysis of protein coexpression modules. A total of 224 protein coexpression modules were identified, with the top modules (C1 - 2, C1 - 3, C1 - 4, C1 - 5, C1 - 26, and C1 - 27) highlighted in the sunburst plot. Module C1 - 2 (46.8%) includes 18 submodules and is enriched in stress response, ribonucleoprotein biogenesis, and neutrophil degranulation pathways. Module C1 - 3 (5.6%) is associated with extracellular matrix and ossification-related pathways. Module C1 - 4 (47.6%) contains branches C1 – 26 and C1 - 27, both enriched in RNA metabolism, stress response, and cell cycle, with no overlapping proteins between them, indicating distinct molecular features. Each node is a cluster identified by multiscale clustering, where the node size is proportional to the cluster size and the node color corresponding to the enriched pathways of the involved proteins.

To obtain a comprehensive understanding of the regulatory network architecture, the Multiscale Embedded Gene Co-expression Network Analysis (MEGENA) method was employed to systematically uncover key regulators (**Figure 4**). A total of 224 tightly interconnected protein coexpression network modules were identified. Among these, the top modules—C1-2, C1 - 3, C1 - 4, C1 - 5, C1 - 26, and C1 - 27—each accounted for more than 10% of the network and were labeled and color-coded in the sunburst plot for visual clarity.

C1-2, accounting for 46.8%, is composed of 18 modules, with C1 – 5 identified as its dominant submodule. Pathway enrichment analysis revealed that proteins within C1 – 2 were primarily involved in cellular responses to stress, ribonucleoprotein complex biogenesis, and neutrophil degranulation.

C1-3, the smallest of the major clusters (5.6%), was made up of two modules. Consistent with cellular component analysis (**Supplementary Figure S2A**), the majority of proteins within C1 – 3 were associated with the extracellular matrix. Furthermore, pathways related to ossification and skeletal system development were enriched in C1 - 3.

C1-4, accounting for 47.6% of the network, represented the largest overall component and included two notable branches, C1 – 26 and C1 - 27. These branches were similarly enriched for pathways related to the metabolism of RNA, cell response to stress, and the cell cycle. Interestingly, no overlapping proteins were found between C1 – 26 and C1 - 27 (**Supplementary Figure S2B**), indicating distinct molecular specificities despite their functional similarities.

### 3.5 Potential hub proteins involved in regulating pathogenesis

**Figure 5:**
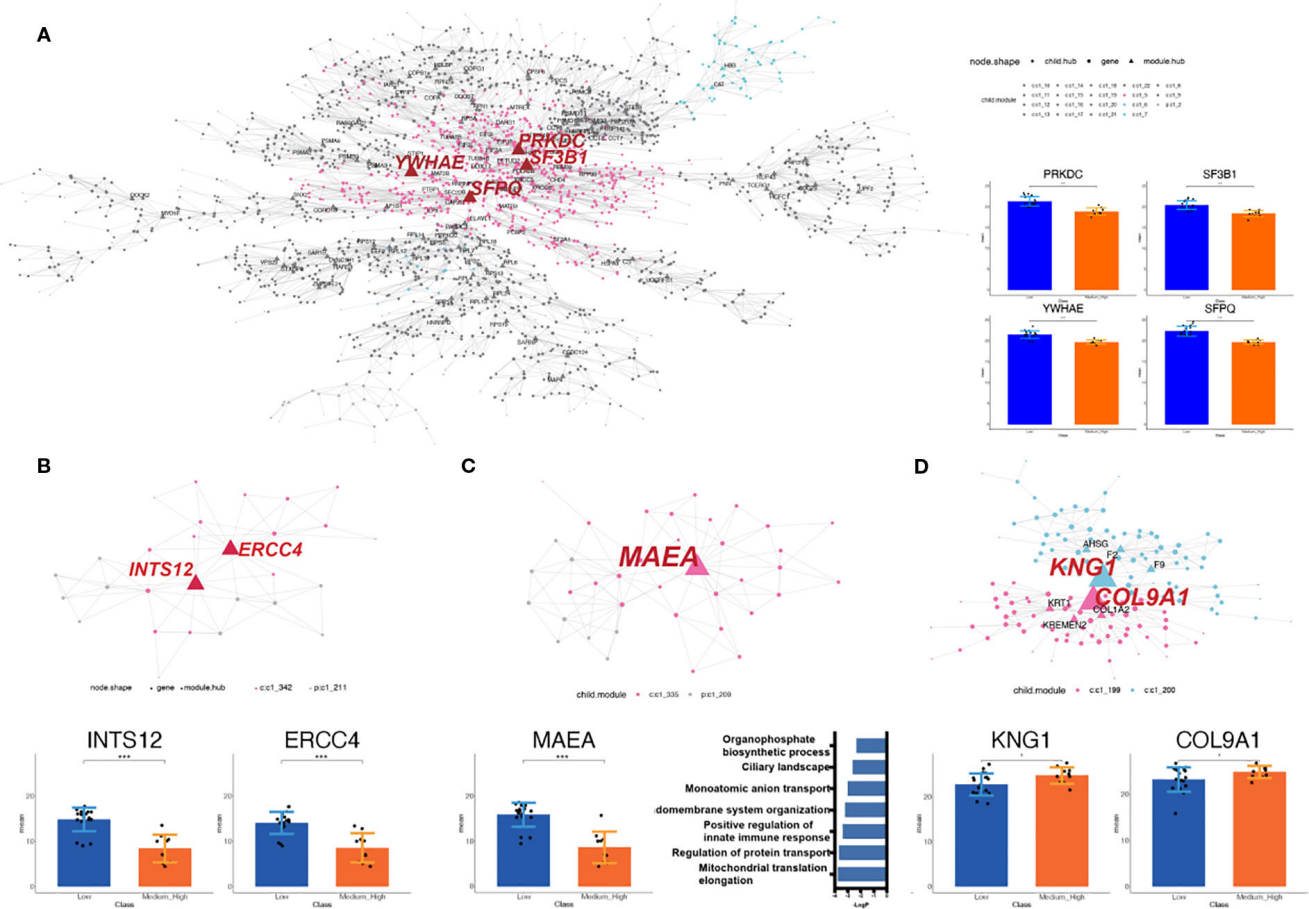
Identification of the potential functional cluster by MEGENA analysis. **(A)** Module C1 - 2, the largest module, contains 2,026 proteins and 110 hubs; top hubs include PRKDC, SF3B1, SFPQ, and the unique hub YWHAE. The nodes with labels are the selected hubs of the cluster (left). The histogram shows the intensity of selected hubs in LNRg vs. MHNRg (right). **(B)** Module C1 – 211 has all proteins significantly differentially expressed, with INTS12 and ERCC4 as key regulators. The nodes with labels are the selected hubs of the cluster (top). The histogram shows the intensity of selected hubs (bottom). **(C)** Modules C1 – 209 and C1 – 335 share the hub gene *MAEA*. The nodes with labels are the hubs of the cluster (top). The histogram shows the intensity of selected hubs (bottom and left). Pathway analysis by Metascape demonstrates significantly involved pathways. The *x*-axis indicates −log10 (*p*-value). **(D)** Modules 199 and 200, enriched in MHNRg, feature hubs KNG1 and COL9A1 with higher expression. The nodes with labels are the hubs of the cluster (top). The histogram shows the intensity of selected hubs. Statistical significance: ^*^ *p*-value < 0.05; ^**^ *p*-value < 0.01; ^***^ *p*-value < 0.001.

Module C1 - 2, the largest identified module, comprised 2,026 proteins (**Supplementary Figure S3A**). Within this module, 110 hub proteins were identified. Notably, 68.2% of the tested proteins give rise to observable altered phenotypes when perturbed by using the mutant phenotype data from the Mouse Genome Informatics database (MGI) (28) (**Supplementary Table S1**). Among them, the top three proteins ranked by the number of regulated targets were Protein Kinase, DNA-Activated, Catalytic Subunit (PRKDC) (38), Splicing Factor 3b Subunit 1 (SF3B1) (36), and Splicing Factor Proline And Glutamine Rich (SFPQ) (34) (**Figure 5A**). In addition, hub gene Tryptophan 5-Monooxygenase Activation Protein Epsilon (YWHAE) was uniquely present in module 2 (**Figure 5A**).

To further clarify the biomolecular alterations associated with osteosarcoma malignancy, we focused on the gene coexpression network module enriched in DEPs. Modules selected for downstream analysis were required to meet the following criteria: (1) a total gene count exceeding 20, (2) at least 85% of the proteins in the module classified as DEPs (defined by fold change > 1.2 or < 0.83), and (3) hub proteins exhibiting fold changes > 1.5, between LNRg and MHNRg, a threshold indicative of strong regulatory potential. In total, 21 modules met these criteria (**Supplementary Figures S3B, C**).

Among these, module C1 – 211 is the only module in which all constituent proteins were significantly differentially expressed between the two groups. Integrator Complex Subunit 12 (INTS12) and ERCC Excision Repair 4, Endonuclease Catalytic Subunit (ERCC4) were analyzed as the potential regulatory proteins in this module (**Figure 5B**). Meanwhile, modules C1 – 209 and C1 - 335, both derived from C1 - 4 (**Supplementary Figure S3D**), shared the same hub gene, MAEA, which clearly distinguished LNRg from MHNRg (**Figure 5C**). The enriched pathway of these modules comprised different categories, including mitochondrial translation elongation, positive regulation of innate immune response, and organophosphate biosynthetic process, suggesting a broad regulatory role for MAEA.

In addition, modules C1 – 199 and C1 – 200 were the only modules characterized by the accumulation of DEPs exclusively in MHNRg. The hub genes in these modules, KNG1 and COL9A1, exhibited significantly higher expression in MHNRg (**Figure 5D**), indicating their potential utility as diagnostic biomarkers predictive of a favorable response to chemotherapy.

### 3.6 Immunohistochemistry verification of protein changes typical for OS development

**Figure 6:**
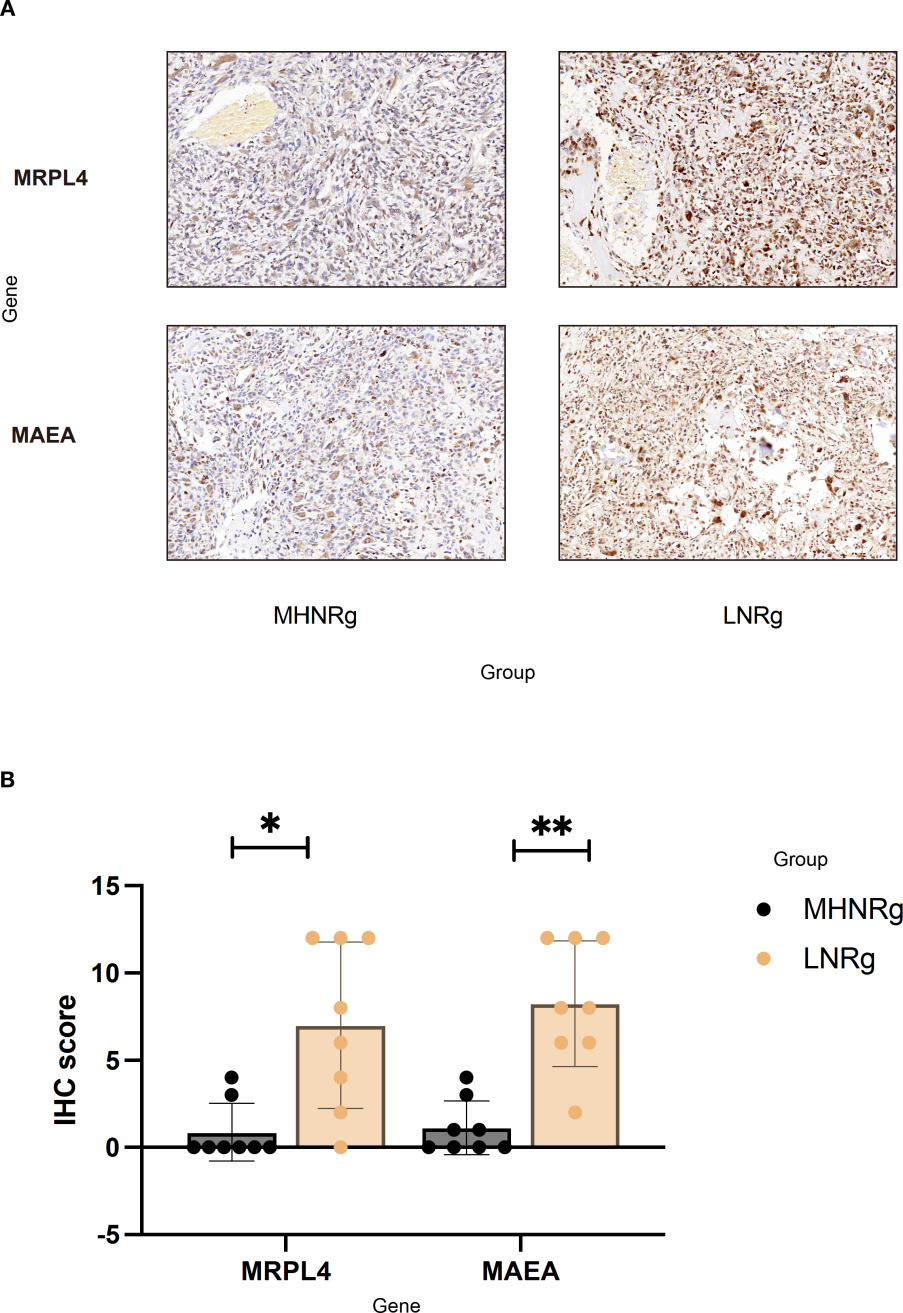
Immunohistochemical verification of protein expression associated with osteosarcoma development. **(A)** Representative staining images of MRPL4 and MAEA in tissue samples from LNRg and MHNRg (× 200 magnification). **(B)** Quantification showing significantly higher expression of MRPL4 and MAEA in LNRg compared to MHNRg. Data are shown as mean ± SD, *N* = 8. ^*^ *p*-value < 0.05; ^**^ *p*-value < 0.01.

To elucidate the potential significance of the selected proteins, immunohistochemical staining was performed for CBX5, MAEA, MRPL4, and MRPL58 on tissue samples from seven high/medium-OS patients, seven low-OS patients, and a control group of seven healthy individuals. Representative staining patterns of MRPL4 and MAEA are shown in **Figure 6A**. Notably, the expression levels of MRPL4 and MAEA were significantly higher in the LNRg than in the MHNRg (**Figure 6B**). Additionally, CBX5 and MRPL58 exhibited higher, though not statistically significant, expression in the LNRg group compared to the MHNRg group (**Supplementary Figure S4A**). These findings suggest that these proteins may serve as potential biomarkers for stratifying necrosis levels and distinguishing disease states in osteosarcoma.

### 3.7 Regulatory network

The coordinated action of multiple proteins and pathways affects the transformation of healthy cells into tumor cells. Runx1 is an important transcription factor that regulates the fate of osteosarcoma stem cells, maintaining the undifferentiated state of osteosarcoma stem cells by collaborating with the Notch pathway. Notch-related signals activate HES1 through DLL1-Notch1/2-mediated ligand-receptor binding between tumor cells, further regulating the expression of differentiation-inhibitory genes. The interplay between these pathways enhances the tumor’s invasiveness and treatment resistance.

In the context of metabolic reprogramming, the PI3K/AKT/mTOR pathway promotes fatty acid metabolism and energy reprogramming by activating FABP4 (fatty acid binding protein) and PLIN1 (lipid droplet protein). The upstream MAPK pathway activates downstream ERK through KRAS and BRAF, and collaborates with the PI3K/AKT pathway to enhance the proliferation of tumor cells. Together, these pathways enhance the adaptability of osteosarcoma cells to high metabolic demands.

Wnt/β-catenin signaling activates CTNNB1 (β-catenin) through WNT1, promoting the expression of collagen (such as COL1A1 and COL28A1), thereby increasing the adhesion and matrix invasion ability of tumor cells. The TGF-β/SMAD pathway, initiated by TGFB1, activates SMAD3, promoting ECM generation and remodeling, which facilitates tumor metastasis. These two pathways synergistically remodel the extracellular matrix, enabling osteosarcoma cells to breach physical barriers and invade surrounding tissues.

NF-κB and immune microenvironment-related pathways are crucial in immune escape and inflammation regulation. The NF-κB signaling pathway activates proinflammatory signals (such as TNF) through IL17RE and CRP, thereby promoting tumor growth in an inflammatory environment.

C4BPB and FCN3 regulate the complement system, inhibit complement-mediated cytotoxicity, and contribute to immune escape. This mechanism makes osteosarcoma more invasive in an inflammatory-rich and immunosuppressive microenvironment.

**Figure 7:**
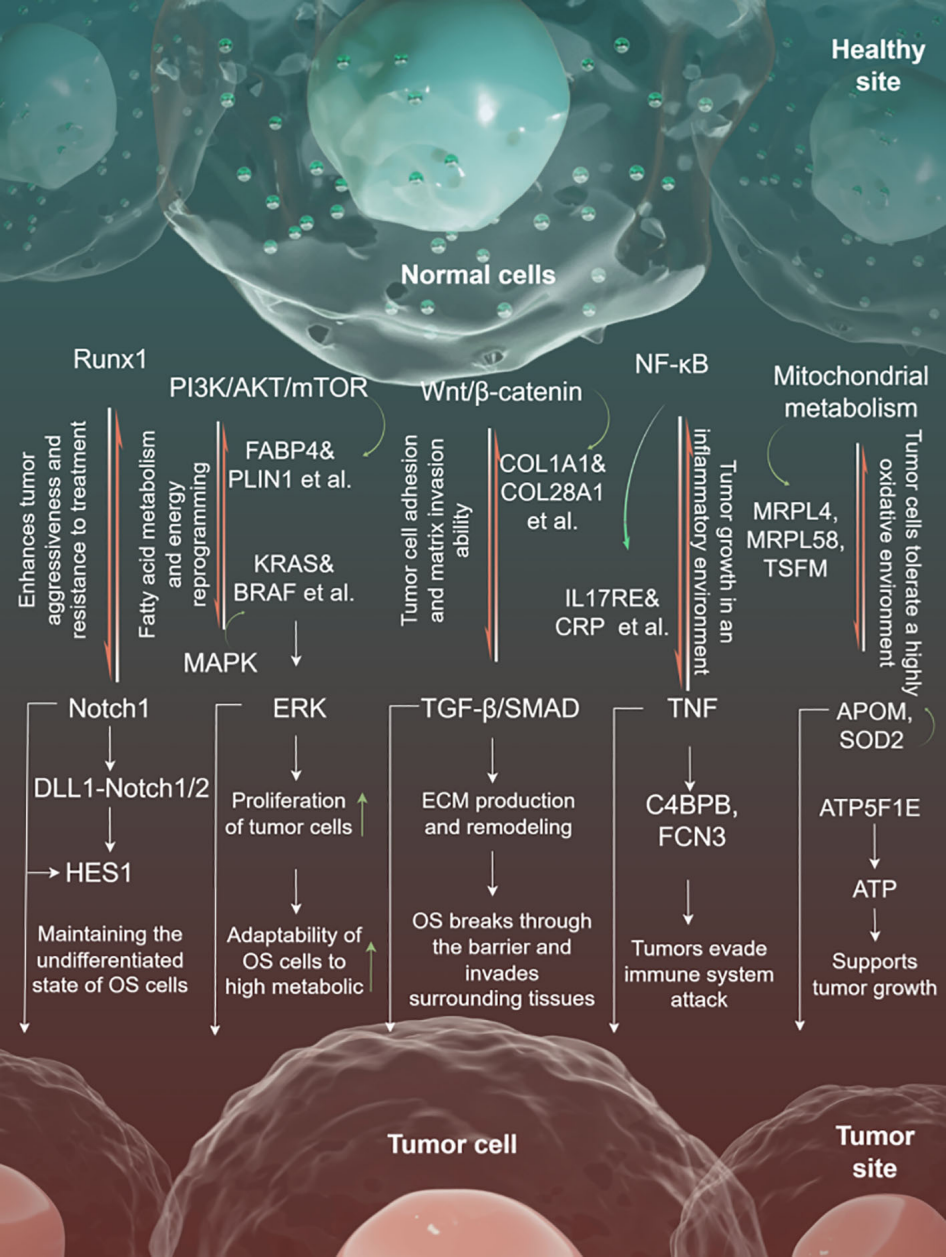
The regulatory mechanism diagram.

The mitochondrial metabolic pathway regulates mitochondrial ribosomal protein synthesis through MRPL4, MRPL58, and TSFM, enhances mitochondrial oxidative phosphorylation capacity, and provides continuous energy for tumor cells. APOM and SOD2 are involved in antioxidant defense, helping tumor cells survive in highly oxidative conditions. ATP5F1E facilitates ATP production and supports tumor proliferation under energy-deficient conditions (**Figure 7**).

## 4 Discussion

OS is a highly malignant primary bone tumor characterized by rapid progression and a high risk of metastasis, posing a serious threat to patients’ survival rates and quality of life (29). Although current multimodal treatment options, including surgery and neoadjuvant chemotherapy, provide certain survival advantages for OS patients, the treatment outcomes remain unsatisfactory due to chemotherapy resistance (30). Studies have found that the tumor necrosis rate caused by chemotherapy is closely related to the patient’s survival rate, suggesting that the molecular mechanisms of tumor response to chemotherapy may play a key role in the progression and treatment of OS (31). Therefore, identifying the potential molecular characteristics of different chemotherapy response groups and exploring their feasibility as therapeutic targets is of great significance for improving the prognosis of OS patients and formulating personalized treatment strategies. The proteomic profiles analyzed in this study were obtained from resected tumor tissue collected after patients had undergone three to four cycles of neoadjuvant chemotherapy, which included methotrexate, ifosfamide, and doxorubicin. Therefore, these profiles represent the tumor state at an intermediate point during chemotherapy and reflect the necrosis response induced by the treatment. While the ideal scenario for assessing inherent pretreatment differences between LNRg and MHNRg patients would involve diagnostic biopsy samples prior to any chemotherapy, the practical constraints of this retrospective study—including the scarcity, limited quantity, and variable preservation quality of such pretreatment biopsies suitable for proteomics—precluded their use. Our primary objective, however, was explicitly focused on identifying proteomic characteristics associated with the achieved level of chemotherapy-induced tumor necrosis, a critical prognostic factor. Analyzing the proteome within the postchemotherapy surgical specimens, in which the necrosis rate was histologically quantified, was therefore essential and directly aligned with this specific research aim. The proteomic differences we report herein are thus interpreted within the context of the tumor’s response to chemotherapy at the time of resection.

This study demonstrated proteomic differences between LNRg and MHNRg using PCA and clustering methods (K-means and hierarchical clustering). These findings support the critical role of the chemotherapy-induced tumor necrosis rate in patient prognosis and suggest that the necrosis rate, as an important efficacy indicator, may provide a basis for improving treatment strategies. The proteins upregulated in MHNRg are concentrated in functional areas such as immune response, oxidative detoxification, lipid metabolism, and collagen formation, suggesting that this group may mobilize immune and metabolic mechanisms more strongly during chemotherapy (32). However, significantly more proteins and higher levels were expressed in LNRg, reflecting possible unique metabolic stress and protein regulation patterns in patients with a poor response to chemotherapy. Functional analysis of upregulated proteins in LNRg revealed enrichment of DNA replication, metabolism, and mitochondria-related pathways. In particular, metabolic reprogramming plays a key role in tumor cell survival and chemotherapy resistance (33). In addition, the involvement of the classic Notch and MAPK signaling pathways further supports the central role of these proteins in the pathogenesis and treatment response of OS (25). FAM180A is identified as a potential marker for tumor microenvironment phenotype prediction and is closely linked to personalized prognosis (34). Combined with the discovery of immune-related proteins (such as IL17RE and PF4) and metabolism-related proteins, these molecules may provide new directions for improving the precise diagnosis and treatment of OS (35). In MHNRg, upregulated immune-related proteins may indicate a more active immune component in the tumor microenvironment of these patients. The enhancement of immune response may have a double-edged sword effect on chemotherapy resistance, which may promote tumor cell clearance or enhance tumor survival through immune evasion (36).

The metabolic processes of LNRg exhibit characteristic findings. Enrichment analysis showed that metabolism-related DEPs in LNRg are involved in a variety of important biological processes. Glycolipid catabolism involving proteins such as GM2A, NAGA, and SGSH is more active in patients with a low necrosis rate, which may be closely related to the energy metabolism needs of tumor cells (37). The biosynthesis of organophosphates mediated by proteins such as INPP4A and PNPO suggests that the active phospholipid metabolism and membrane dynamics regulation in LNRg samples are enriched through folate metabolism mediated by proteins such as DHFR, indicating a possible increase in the demand for nucleic acid synthesis, consistent with the characteristics of rapidly proliferating tumor cells (38). These metabolic activities indicate that there is specific metabolic adaptability in LNRg samples, thereby supporting tumor survival and chemotherapy resistance. The 15 mitochondria-related DEPs highlight their central role in tumor metabolism, regulating molecular mechanisms such as mitochondrial translation elongation factor (TSFM) and mitochondrial ribosomal proteins (MRPL series), enhancing the energy production capacity of mitochondria and supporting the proliferation needs of tumor cells in a low necrosis rate environment. Enhanced mitochondrial function is often associated with tumor cells’ tolerance to oxidative stress, increased metabolic flexibility, and chemotherapy resistance (39). TSFM and MRPL series proteins, as the core of mitochondrial protein synthesis and metabolic regulation, directly affect the functional integrity of mitochondria and tumor metabolic needs. The hub status of MRPL4 and MRPL58 indicates that they may have the ability to regulate the expression and function of other metabolism-related proteins in the network (40). Data integration analysis further verified the potential importance of MRPL4. The high expression of MRPL4 in OS samples, especially in metastatic samples, suggests that it may promote tumor invasiveness by enhancing mitochondrial metabolic activity and energy supply (41).

The Runx1 signaling pathway plays a role in the development of OS by supporting active metabolism, regulating the cell cycle, and inhibiting ossification differentiation (42). It is positively correlated with metabolic pathways and may help tumors adapt to microenvironmental stress by regulating metabolic needs. By regulating cell cycle-related proteins and promoting rapid cell proliferation, it provides a material basis for the aggressive characteristics of OS (43). The negative correlation with the ossification pathway indicates that Runx1 signaling may hinder the differentiation of tumor cells, allowing them to maintain a malignant state of high proliferation and low differentiation (44). This model reveals the important role of Runx1 in tumor proliferation and metabolic adaptation and suggests that it may inhibit ossification differentiation, thus maintaining the proliferative and undifferentiated state of tumor cells. It highlights the versatility of Runx1 as a potential tumor regulator, engaging in processes that directly influence OS aggressiveness and therapeutic response. The expression of multiple key proteins involved in the Runx1-related pathway (RING1, SMARCB1, ARID1A, etc.) was significantly higher in LNRg samples than in MHNRg. As the central node of the PPI network, HDAC1 has a synergistic effect with SMARCB1, UBQLN2, ENY2, and BAZ1B. As part of the Runx1 pathway, these proteins not only play roles in signaling and chromatin remodeling but may also regulate metabolism and antiapoptotic mechanisms, enhancing the survival ability of OS cells (45). HDAC1, UBQLN2, and BAZ1B, among others, are highly expressed in OS samples and are significantly associated with metastasis, suggesting that they may serve as potential biomarkers for diagnosis and prognosis. The pivotal role of HDAC1 makes it an attractive therapeutic target, and HDAC inhibitors may inhibit tumor growth and metastasis by interfering with chromatin remodeling and gene expression regulation (46).

MEGENA analysis provides a comprehensive picture of the OS regulatory network, revealing multiple key modules and their potential connections to disease mechanisms. The distribution of top modules and their enriched pathways provides important clues for understanding the pathological mechanisms of OS. The C1 – 2 module is distinguished by its regulation of cellular stress responses and ribonucleoprotein complex biosynthesis, which are closely related to the ability of tumor cells to adapt and maintain survival in harsh microenvironments. The enrichment of neutrophil degranulation implicates the role of inflammation in tumor development and may influence immune regulation of the tumor microenvironment, which further highlights the potential critical role of the C1 – 2 module in OS progression (47). In contrast, although the C1 – 3 module is the smallest, its functional characteristics are highly targeted. The proteins of this module are mainly concentrated in the extracellular matrix and participate in pathways related to ossification and skeletal system development, which are directly related to the tissue origin and aggressive characteristics of OS. These results suggest that the C1 – 3 module may play a more defined pathological role in OS, specifically related to the interaction between tumor cells and their stroma. The C1 – 4 module accounts for the largest proportion in the network, indicating its central position in the OS regulatory network. Its enriched pathways focus on RNA metabolism, cellular stress response, and cell cycle, which are basic functions required for the rapid proliferation of tumor cells (48). However, the lack of common proteins between the two branches, C1 – 26 and C1 - 27, shows functional specificity within the module, which reflects differences in molecular mechanisms at different stages or states in tumor progression. This functional specificity not only demonstrates the complexity of the module but also provides potential specific targets for subsequent studies aiming at therapeutic intervention targeting different subtypes or stages of development.

Regulatory hub proteins in module 2 revealed the broad roles of key molecules in OS pathogenesis, with PRKDC, SF3B1, and SFPQ ranking at the top in terms of the number of regulatory targets, indicating their central position in the regulatory network. PRKDC is closely related to DNA damage repair, and its high regulation may reflect the adaptive strategy of OS cells in response to genome instability, while the functions of SF3B1 and SFPQ are related to RNA splicing and gene expression regulation, further indicating that these genes play a key role in the rapid proliferation and metastasis of OS (49). INTS12 and ERCC4 in module 211 are cosignificantly expressed proteins in both LNRg and MHNRg, highlighting their prevalence and key role in different necrosis rate groups, especially their association with DNA damage response, which may have significant implications for malignant OS (50, 51).

The role of MAEA in chemotherapy resistance is evidently context-dependent and multifaceted. As a core component of the GID/CTLH E3 ubiquitin ligase complex, MAEA promotes cell proliferation by targeting critical transcription factors such as HBP1 for ubiquitination and subsequent proteasomal degradation, underscoring its key regulatory functions in tumorigenesis (52). Our bioinformatic analyses, coupled with immunohistochemical validation, further establish the clinical relevance of MAEA, causing poor response to chemotherapy, as demonstrated by its significantly elevated expression in LNRg tissue samples. Consistent with these findings, MAEA-mediated ubiquitination and degradation of PHD3 has been shown to facilitate glioblastoma progression and confer resistance to temozolomide treatment, highlighting its role in chemoresistance mechanisms in this context (53). Intriguingly, in gastrointestinal cancers, MAEA overexpression paradoxically enhances chemosensitivity via promoting PARP1 ubiquitination and degradation, suggesting that MAEA’s influence on chemotherapy response is tumor type- and context-specific (54).

Mechanistically, our study identifies MAEA as a hub gene within modules 209 and 335, implicating its involvement in regulating mitochondrial translation elongation and innate immune responses. These processes collectively support metabolic reprogramming and create an immunosuppressive tumor microenvironment that favors survival during cytotoxic stress (55, 56). Moreover, MAEA has been implicated in promoting autophagy, a process known to contribute to tumor cell survival and drug tolerance (57). These findings suggest that MAEA facilitates a multifactorial adaptation of tumor cells to chemotherapy through metabolic flexibility and immune evasion.

Furthermore, the potential crosstalk between MAEA and RUNX family transcription factors is highlighted. RUNX2 is recognized for its role in bone sarcomas, where it regulates bone turnover and influences response to targeted therapies (58). Given RUNX2’s involvement in transcriptional networks associated with metabolism and tumor progression, it may serve as an upstream regulator of MAEA or its related pathways, thereby modulating chemotherapy resistance in tumors such as osteosarcoma. This potential regulatory axis warrants further investigation to clarify the molecular mechanisms through which RUNX-related pathways influence MAEA-mediated metabolic and immune responses in the context of poor response to chemotherapy.

Considering the low incidence of osteosarcoma, we acknowledge that the limited sample size remains a key limitation of this study. The current dataset reflects a 5-year accumulation of cases in our center, and increasing the cohort size would improve the robustness and statistical power of the analysis, which we intend to pursue in future research. Although the expression differences of CBX5 and MRPL58 were not statistically significant, their importance in the network and consistent protein expression trends highlight the need to further investigate their functions. These findings confirm the value of regulatory networks in guiding disease stratification and mechanistic research, while also providing new perspectives for the future development of therapeutic strategies targeting specific molecular mechanisms.

This study systematically analyzed the proteomic characteristics associated with differences in necrosis rates among OS patients and revealed key molecular mechanisms underlying OS progression and metastasis. Differences in metabolic characteristics among necrosis rate groups were found to involve not only mitochondrial function and metabolic activity but also specific modules related to cell cycle regulation and immune response. In addition, a series of potential hub proteins and regulatory modules were identified through MEGENA network and protein–protein interaction analysis, and the important roles of key proteins such as MRPL4 and MAEA in distinguishing necrosis rates were confirmed by immunohistochemistry. These findings provide new insights into the relationship between the metabolic characteristics of OS and its pathological processes, offering an important basis for developing personalized diagnostic markers and treatment strategies. Furthermore, they reveal the complex network of OS metabolism and molecular regulation, laying a foundation for precision medicine research.

## Data Availability

The datasets presented in this study can be found in online repositories. The names of the repository/repositories and accession number(s) can be found in the article/**Supplementary Material**.

## Supplementary material

Supplementary Figure 1 Proteomic analysis of the OS clinical specimens. **(A-C)** PCA analysis display proteomic heterogeneity between sex, subtype and pathogenic site; **(D)** Data normalization and estimation for clustering; **(E)** Visualization of cluster profile plot.

Supplementary Figure 2 Analysis for top module by MEGENA analysis. **(A)** Cellular component analysis by Metascape for C1_3; **(B)** Venn diagram analysis of the proteins in C1_26 and C1_27.

Supplementary Figure 3 Selection criteria of the module for biomarker. **(A)** Ranking list of quantities of proteins in top 11 modules; **(B)** The selection criteria of module for biomarker development; **(C)** Dot plot showing 21 modules in **(B)**; **(D)** Sunburst plot showing the relationship.

Supplementary Figure 4 IHC staining of MRPL58 and CBX5 in tissue of human samples. **(A)** IHC scores for MRPL58 and CBX5 in MHNRg and LNRg. Data are shown as mean ± SD, n = 8.
