# Supplementary figures and images for: Identification of MAEA protein as a potential target for chemoresistance in osteosarcoma using bioinformatics and proteomic analysis
Source: Front Oncol. 2025 Sep 8;15:1597750. doi: 10.3389/fonc.2025.1597750 (PMC12450695; doi:10.3389/fonc.2025.1597750)

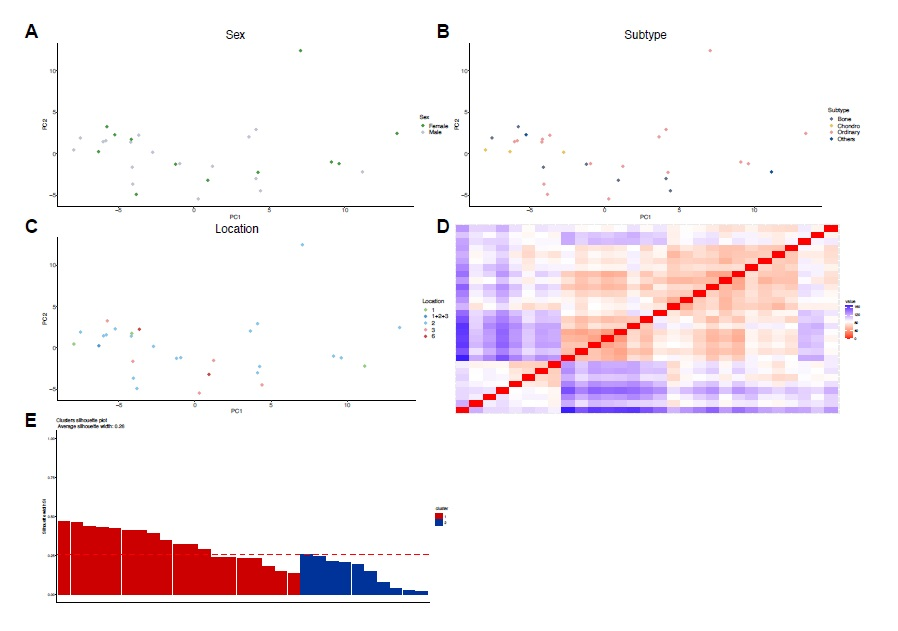

Supplement: Supplementary Figure 1 — Proteomic analysis of the OS clinical specimens. (A-C) PCA analysis display proteomic heterogeneity between sex, subtype and pathogenic site; (D) Data normalization and estimation for clustering; (E) Visualization of cluster profile plot. [file Image1.png]

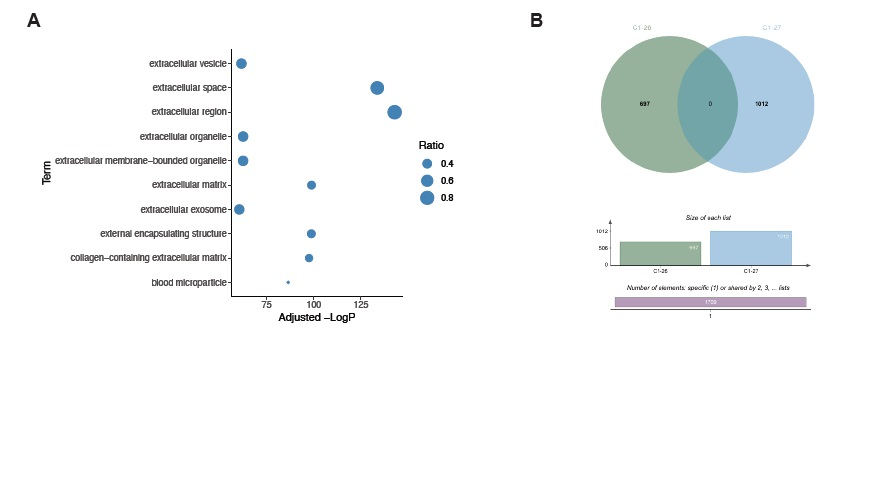

Supplement: Supplementary Figure 2 — Analysis for top module by MEGENA analysis. (A) Cellular component analysis by Metascape for C1_3; (B) Venn diagram analysis of the proteins in C1_26 and C1_27. [file Image2.png]

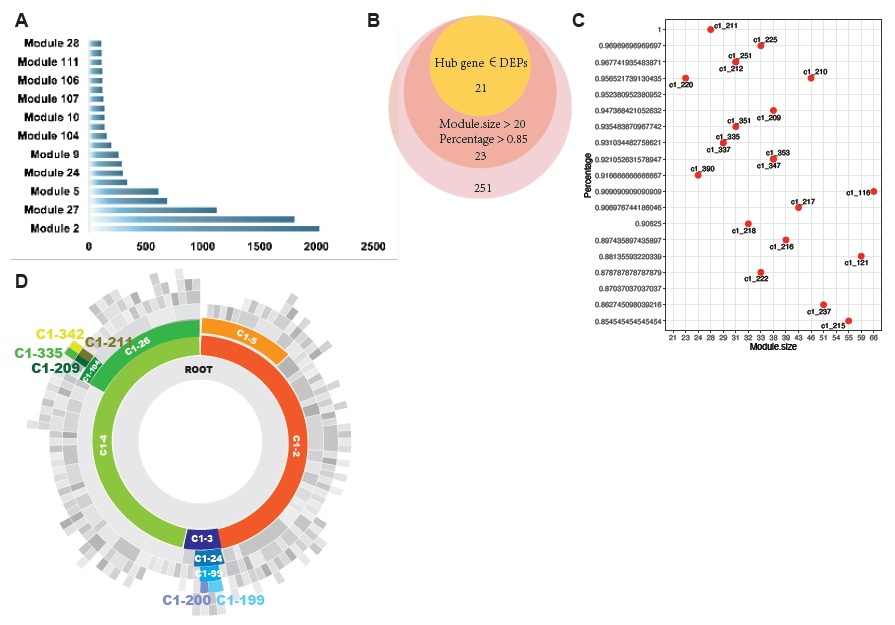

Supplement: Supplementary Figure 3 — Selection criteria of the module for biomarker. (A) Ranking list of quantities of proteins in top 11 modules; (B) The selection criteria of module for biomarker development; (C) Dot plot showing 21 modules in (B); (D) Sunburst plot showing the relationship. [file Image3.png]

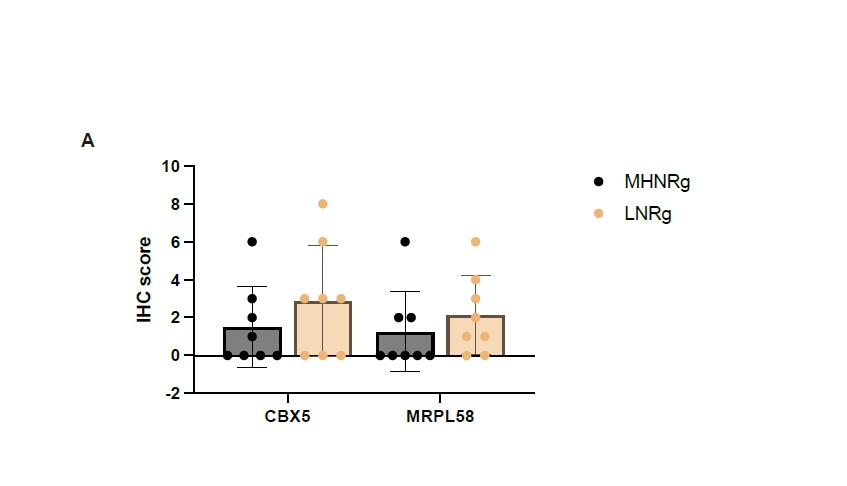

Supplement: Supplementary Figure 4 — IHC staining of MRPL58 and CBX5 in tissue of human samples. (A) IHC scores for MRPL58 and CBX5 in MHNRg and LNRg. Data are shown as mean ± SD, n = 8. [file Image4.png]
